# BrlA and AbaA Govern Virulence-Required Dimorphic Switch, Conidiation, and Pathogenicity in a Fungal Insect Pathogen

**DOI:** 10.1128/mSystems.00140-19

**Published:** 2019-07-09

**Authors:** An-Xue Zhang, Amina-Zahra Mouhoumed, Sen-Miao Tong, Sheng-Hua Ying, Ming-Guang Feng

**Affiliations:** a MOE Laboratory of Biosystems Homeostasis & Protection, College of Life Sciences, Zhejiang University, Hangzhou, Zhejiang, China; b College of Agricultural and Food Science, Zhejiang A&F University, Lin’an, Zhejiang, China; Vanderbilt University

**Keywords:** blastospore formation, conidiation, developmental activators, dimorphic transition, entomopathogenic fungi, pathogenicity, virulence

## Abstract

Dimorphic plant and human mycopathogens require a switch from the usual yeast growth to filamentous growth for host tissue penetration, and the switch is controlled by multiple signaling systems other than the central developmental pathway. Unlike these fungi, dimorphic insect mycopathogens usually grow by hyphal extension, infect the host by hyphal penetration through the insect cuticle, and switch to unicellular blastospores from the penetrating hyphae only after entry into the host hemocoel, where blastospore propagation by yeast-like budding accelerates host mummification. Here, we report a dependence of the virulence-required dimorphic transition on the central pathway activators BrlA and AbaA in Beauveria bassiana. Deletion of *brlA* or *abaA* abolished both aerial conidiation and submerged blastospore formation *in vitro* despite no negative impact on hyphal growth in various media, including a broth mimic of insect hemolymph. The hyphae of either deletion mutant lost insect pathogenicity through normal cuticle penetration, contrasting with a high infectivity of wild-type hyphae. The mutant hyphae injected into the host hemocoel failed to form blastospores, resulting in slower lethal action. Uncovered by transcriptomic analysis, several genes involved in host adhesion and cuticle degradation were sharply repressed in both deletion mutants versus wild type. However, almost all signaling genes homologous to those acting in the dimorphic switch of other fungi were not differentially expressed at a significant level and hence unlikely to be involved in shutting down the dimorphic switch of each deletion mutant. Therefore, like aerial conidiation, the submerged dimorphic switch *in vitro* and *in vivo* is a process of asexual development governed by the two central pathway activators in B. bassiana.

**IMPORTANCE** Dimorphic insect mycopathogens infect the host by hyphal penetration through the host cuticle and switch from the penetrating hyphae to unicellular blastospores after entry into the host hemocoel, where blastospore propagation by yeast-like budding accelerates host mummification to death. The fungal virulence-required dimorphic switch is confirmed as a process of asexual development directly regulated by BrlA and AbaA, two key activators of the central developmental pathway in an insect mycopathogen. This finding unveils a novel mechanism distinct from the control of the dimorphic switch by multiple signaling systems other than the central developmental pathway in dimorphic plant and human mycopathogens, which switch from the usual yeast growth to filamentous growth required for pathogenicity through host tissue penetration.

## Abstract (precis)

Dimorphic insect mycopathogens infect the host by hyphal penetration through the host cuticle and switch from the penetrating hyphae to unicellular blastospores after entry into the host hemocoel, where blastospore propagation by yeast-like budding accelerates host mummification to death. The fungal virulence-required dimorphic switch is confirmed as a process of asexual development directly regulated by BrlA and AbaA, two key activators of the central developmental pathway in an insect mycopathogen. This finding unveils a novel mechanism distinct from the control of the dimorphic switch by multiple signaling systems other than the central developmental pathway in dimorphic plant and human mycopathogens, which switch from the usual yeast growth to filamentous growth required for pathogenicity through host tissue penetration.

## INTRODUCTION

Beauveria bassiana is a filamentous fungal insect pathogen that usually undergoes an asexual cycle and displays either a saprophytic or an entomopathogenic lifestyle (1). As a classic biological control agent for insect pests, B. bassiana starts host infection by conidial adhesion to the host cuticle, where conidial germination leads to hyphal extension for penetration through the host cuticle by means of the actions of various cuticle-degrading enzymes (2). Upon entry into the host hemocoel, the penetrating hyphae turn into unicellular blastospores (also called hyphal bodies) that are enabled to propagate rapidly by yeast-like budding until the host is mummified to death (3–6). At or near the time of host death, intrahemocoel hyphal bodies turn back into septate hyphae to penetrate the host cuticle again for outgrowth (7, 8). Ultimately, aerial conidia are produced on the surfaces of host cadavers for survival and dispersal for a new infection cycle. The process of dimorphic transition, by which penetrating hyphae turn into hyphal bodies and vice versa, is critical for the virulence and asexual cycle *in vivo* of a fungal insect pathogen, although the mechanisms involved remain poorly understood. This process has been evidently associated with the functions of many genes characterized in B. bassiana, such as those encoding the histone acetyltransferases Gcn5 and Mst2 (7, 9), the histone deacetylases Hos2 and Rpd3 (10, 11), the mitogen-activated protein kinase (MAPK) kinase Ste7 (12), the Na^+^/H^+^ antiporter Nhx1 (13), the blue-light photoreceptor VVD (6), the vacuolar protein VLP4 (14) or ATPase subunit H VmaH (15), the lysyl-tRNA synthetase KRS (16), and the cyclophilin B CypB (17). Interestingly, these studied genes are also responsible for a correlation of dimorphic transition *in vitro* or *in vivo* with aerial conidiation and transcriptional activation of key activator genes in the central development pathway, as summarized in Table 1. Thus, the previous studies suggest a closer linkage of the dimorphic switch with the developmental activators in *B.* bassiana than the signaling factors involved in two-component and heterotrimeric G protein signaling systems and the Ras and cyclic AMP (cAMP) signaling and the downstream MAPK signaling cascades, all of which are considered to control the dimorphic switch and pathogenicity in human mycopathogens (18).

**TABLE 1:** Case studies for a linkage of dimorphic transition with conidiation and/or expression of key developmental activators in *B.* bassiana

Gene mutant	Gene annotation	Dimorphic transition^*a*^		Conidiation and yield loss (%)^*b*^	Repression of *brlA*, *abaA*, and *wetA*, respectively (%)^*c*^	Reference
		Status *in vivo*	% decrease *in vitro* (medium)			
Δ*gcn5*	Histone acetyltransferase Gcn5	SB	92 (T)	SD, 97	99, 89, 84	8
Δ*mst2*	Histone acetyltransferase Mst2	NE	65 (T)	SD, 75	94, 72, 75	9
Δ*hos2*	Histone deacetylase Hos2	NE	32 (C), 52 (T)	SD, 76	94, 88, 68	10
Δ*rpd3*	Histone deacetylase Rpd3	SB	93 (C), 95 (T)	ESD, 97	94, 98, 57	11
Δ*ste7*	Fus3-cascaded MAPK kinase	NE	68 (T)	SD, 77	84, 90, 97	12
Δ*vvd*	Blue-light photoreceptor VVD	SB	40 (C), 97 (T)	ESD, 60	90, 94, 75	6
Δ*krs*	Lysyl-tRNA synthetase KRS	SB	58 (C), 71 (T)	SD 20	80, 81, 77	16
Δ*VLP4*	Vacuole-localized protein 4	SB	99 (T)	ESD, 80	99, 99, 94	14
Δ*cypB*	Cyclophilin B (CypB)	NE	48 (C), 83 (T)	SD, 47	97, 95, 75	17
Δ*vmaH*	Vacuolar ATPase subunit H	NE	60 (T)	ESD, 40	NE	15
Δ*nhx1*	Na^+^/H^+^ antiporter Nhx1	SB	72 (C), 86 (T)	ESD, 85	NE	13
Δ*GEL1*	Gelsolin GEL1	NE	90 (S)	ESD, 68	NE	7
Δ*mas5*	DnaJ protein Mas5	SB	59 (S)	ND, 50	NE	4

a The status of dimorphic transition *in vivo* (SB, severely blocked; NE, not examined) was revealed through microscopic examination of hemolymph samples taken from Galleria mellonella larvae surviving a period after topical application for normal cuticle infection or intrahemocoel injection for cuticle-bypassing infection. The decrease of dimorphic transition *in vitro* was quantified from submerged liquid cultures of conidia grown in Sabouraud dextrose broth (S), minimal Czapek broth (C), or trehalose-peptone broth (T) mimicking insect hemolymph, as indicated by the abbreviations in parentheses.

b Conidiation on the standard medium Sabouraud dextrose agar plus yeast extract (SDAY) under the optimal regime of 25°C in a light/dark cycle of 12:12 suffered severe delay (SD), extremely severe delay (ESD), or no delay (ND). The loss of conidial yield was assessed from the cultures at the end of a 7- to 12-day incubation.

c The transcriptional repression of each gene was assessed from the cDNA samples derived from 3-day-old SDAY cultures under the optimal regime. NE, not examined.

The asexual cycles *in vitro* of filamentous fungal pathogens comprise distinct phases, including vegetative (hyphal) growth, conidiophore development, and conidiation. Hyphal growth starts from conidial germination, forming germ tubes for hyphal extension. After a period of hyphal growth, some hyphal cells differentiate into conidiogenic cells for formation of the conidiophores to support conidial production. This conidiation process is a cellular event precisely timed and genetically programmed in response to internal and external cues and is genetically controlled by the central developmental pathway consisting of the activators BrlA, AbaA, and WetA, which have been well characterized in *Aspergillus* and *Penicillium* and reviewed elsewhere (19, 20). In filamentous fungi, BrlA, AbaA, and WetA activate the expression of downstream conidiation-specific genes in a hierarchical manner during the development of conidiophores and the formation and maturation of conidia (21, 22). The key activator BrlA is a C_2_H_2_ zinc finger transcription factor that governs the initiation of conidiophore development (23, 24), followed by sequential activation of AbaA in the middle phase of conidiophore development (23, 25, 26) and of WetA in the late phase to activate the expression of proteins or enzymes involved in the synthesis of spore wall components (27–29). Aside from WetA involved in conidial maturation, the velvet protein VosA downstream of the central pathway is required for both the repression of BrlA expression for termination of its control cycle and the biosynthesis of trehalose for conidial maturation (30, 31).

The regulatory role of the central developmental pathway in fungal insect pathogens remains poorly understood. In B. bassiana, loss-of-function mutations of *wetA* and *vosA* have been shown to reduce conidiation capacity by 98% and 88%, respectively; impair cell walls; and decrease intracellular trehalose accumulation (32). However, the upstream key activators BrlA and AbaA have not been functionally explored due to the lack of conidium production by the deletion mutant of either coding gene and an inability to rescue the deleted gene through conventional *Agrobacterium*-mediated transformation with conidia (33). Dimorphic fungi, such as phytopathogenic Ustilago maydis and human-pathogenic Candida albicans, usually grow in a yeast form outside the host and require a switch to filamentous growth for tissue penetration during host infection (34–36). Unlike dimorphic plant and human mycopathogens, filamentous fungal insect pathogens usually grow by hyphal extension outside the host and propagate by yeast-like budding only after entry into the host hemocoel through cuticular penetration. The fungal propagation in the host hemocoel determines the speed of host death from mummification and requires a switch to blastospores from the penetrating hyphae. The opposite direction of the dimorphic switch outside or inside the host suggests a possibly distinct mechanism underlying the dimorphic transition of insect mycopathogens. Since both aerial conidiation and dimorphic transition were closely linked to transcriptional expression of the developmental activator genes (Table 1), we hypothesize that, like aerial conidiation, the submerged dimorphic transition required for fungal virulence could be a process of asexual development to be primarily governed by the central pathway in B. bassiana and other filamentous fungal insect pathogens. This study aims to test the hypothesis. The deletion mutants of *brlA* and *abaA* and their complementary strains constructed with the protoplasts from the hyphal cells of the deletion mutants were analyzed in parallel with the parental wild-type strain. Our findings unveil that BrlA and AbaA act as master regulators of not only aerial conidiation but also insect pathogenicity and dimorphic transition in B. bassiana.

## RESULTS

### Transcriptional profiles and subcellular localization of BrlA and AbaA in B. bassiana.

An online search through the *B.* bassiana genome (37) with the queries of Aspergillus nidulans BrlA and AbaA sequences resulted in identification of orthologous BrlA (EJP63618) and AbaA (EJP70670), which consist of 370 and 901 amino acids with molecular sizes of 41.52 and 100.46 kDa, respectively. The B. bassiana BrlA and AbaA are characterized, respectively, by two C-terminal C_2_H_2_ zinc finger (ZnF) motifs and an N-terminal transcriptional enhancer activator (TEA) motif, both of which are shared by the counterparts of 15 other filamentous, yeast, or yeast-like fungi (see Fig. S1 in the supplemental material). The two orthologs are phylogenetically closer to the counterparts of entomopathogenic fungi, including Cordyceps militaris and Metarhizium spp., than of nonentomopathogenic fungi.

10.1128/mSystems.00140-19.1 FIG S1 Bioinformatic analysis of BrlA (A) and AbaA (B) orthologs found in Beauveria bassiana and some representative fungi. Each fungal name is followed by the NCBI accession code of each protein and its sequence identity to the corresponding ortholog in B. bassiana. Phylogenetic relationships are analyzed using the neighbor-joining method in MEGA7 software at http://www.megasoftware.net/. The bootstrap values of 1,000 replications are given at nodes. Scale, branch length proportional to genetic distance assessed with the neighbor-joining method. The molecular sizes and main motifs of all orthologs are revealed by online structural analysis at https://www.ncbi.nlm.nih.gov/Structure/. Download FIG S1, JPG file, 1.3 MB. Copyright © 2019 Zhang et al. 2019 Zhang et al. This content is distributed under the terms of the Creative Commons Attribution 4.0 International license.

**FIG 1:**
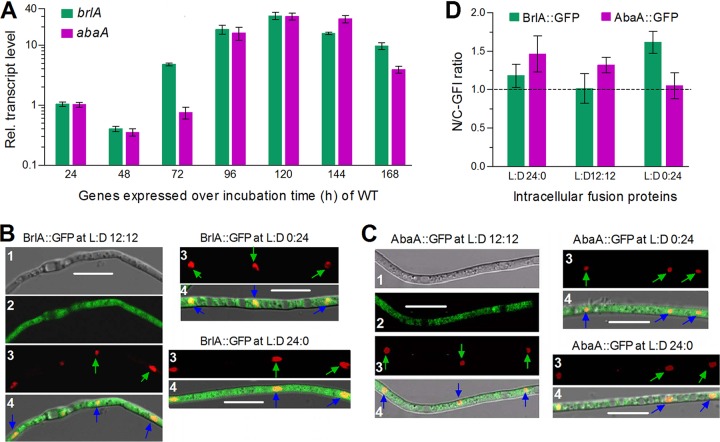
Transcriptional profiles and subcellular localization of BrlA and AbaA in B. bassiana. (A) Relative transcript levels of *brlA* and *abaA* in the WT cultures during a 7-day incubation on SDAY under the optimal regime of 25°C and L:D 12:12 with respect to the standard at the end of the 24-h incubation. (B and C) LSCM images (bars, 10 μm) for subcellular localization of BrlA::GFP (B) and AbaA::GFP (C) fusion proteins expressed in hyphal cells, which were collected from the 3-day-old SDB cultures grown under the optimal regime and stained with DAPI. Bright, expressed (green), DAPI-stained (shown in red), and merged views of the same field are numbered 1, 2, 3, and 4, respectively. Green and blue arrows indicate the nuclei in the stained and merged views, respectively. (D) Ratios of nuclear to cytoplasmic green fluorescence intensities (N/C-GFI) measured from hyphal cells. Error bars, SD of the mean from three cDNA samples analyzed in a qPCR (A) or at least 10 hyphal cells (D).

Transcriptional profiles of *brlA* and *abaA* in the wild-type strain B. bassiana ARSEF 2860 (designated WT here) were monitored during a 7-day incubation of conidia spread on plates of Sabouraud dextrose agar plus yeast extract (SDAY) under the optimal regime of 25°C in a light/dark cycle (L:D) of 12:12 h. Compared to the standard level at the early hyphal extension stage of 24-h incubation, the transcript level of either *brlA* or *abaA* was suppressed during hyphal growth (day 2), followed by a sharp elevation to ∼30-fold on day 5 (Fig. 1A). Notably, the sharp elevation of *brlA* occurred 24 h earlier (day 3) than that of *abaA*, which sustained the transcript peak 24 h longer. These data suggest a 24-h-earlier activation of *brlA* than of *abaA* in the central developmental pathway of B. bassiana.

Laser scanning confocal microscopic (LSCM) analysis of the green fluorescent protein (GFP)-tagged BrlA and AbaA fusion proteins expressed in the WT strain demonstrated a localization of BrlA (Fig. 1B) or AbaA (Fig. 1C) in both the cytoplasm and nucleus of hyphal cells, which were collected from 3-day-old liquid cultures grown in Sabouraud dextrose broth (SDB; i.e., agar-free SDAY) under the optimal regime and stained with a nucleus-specific dye. The subcellular localization of either fusion protein was consistent irrespective of the hyphal cultures grown under continuous light (L:D 24:0) or dark (L:D 0:24) conditions. The ratios of nuclear to cytoplasmic green fluorescence intensities quantified from the hyphal cells also showed a higher accumulation level of each fusion protein in the nucleus than in the cytoplasm at L:D 0:24, 12:12, or 24:0 (Fig. 1D). The nuclear localization indicated a possibility for either BrlA or AbaA to act as a transcription factor.

### BrlA and AbaA are indispensable for conidiation but nonessential for hyphal growth.

Either *brlA* or *abaA* was deleted from WT via homogeneous recombination of its 5′ and 3′ coding/flanking fragments separated by the *bar* marker and rescued by ectopic integration into an identified deletion mutant of the cassette comprising its full-length coding/flanking sequence and the *sur* marker (Fig. S2 and Table S1). As a result, fungal colonies initiated by attaching uniform hyphal mass discs (5-mm diameter) to the plates of rich SDAY, a standard medium for cultivation of entomopathogenic fungi, became fluffier in the Δ*abaA* mutant but showed little morphological change in the Δ*brlA* mutant (Fig. S3A). After a 7-day incubation under the optimal regime, colony sizes were not significantly different between Δ*brlA* and WT strains (Tukey’s honestly significant difference [HSD], *P* > 0.05) irrespective of being grown on SDAY, minimal Czapek agar (CZA), and almost all of 38 CZAs amended with different carbon (sugars/polyols) or nitrogen (inorganic/organic) sources (Fig. S3B). In contrast, the Δ*abaA* mutant grew significantly faster than the control (WT and complementary) strains on most of the minimal media despite moderately decreased or unchanged colony sizes on SDAY and a few CZAs amended with the nitrogen sources NH_4_^+^, NO_2_^−^, tryptophan, and alanine, respectively. These observations indicated a nonessential role for either BrlA or AbaA in vegetative growth of B. bassiana.

10.1128/mSystems.00140-19.2 FIG S2 Generation and identification of B. bassiana *brlA* and *abaA* mutants. (A and B) Diagrams for the strategy for deletion of *brlA* and *abaA* and their mutants identified via PCR (lanes 1 to 3) and Southern blotting (lanes 4 to 6) analyses with paired primers and amplified probe (Table S1). Lanes 1 and 4, wild type; lanes 2 and 5, deletion mutant; lanes 3 and 6, complementary mutant. Genomic DNAs were digested with the enzymes at the marked sites for detection of *brlA* or *abaA* by Southern blot hybridization. Download FIG S2, JPG file, 0.4 MB. Copyright © 2019 Zhang et al. 2019 Zhang et al. This content is distributed under the terms of the Creative Commons Attribution 4.0 International license.

10.1128/mSystems.00140-19.3 FIG S3 Impact of *brlA* or *abaA* deletion on the hyphal growth of B. bassiana. (A) Images of 7-day-old fungal colonies grown at 25°C on plates of rich SDAY and minimal CZA. (B) Diameters of 7-day-old colonies grown at 25°C on plates of SDAY, CZA, and amended CZAs, which were free of carbon (C^−^), nitrogen (N^−^), or both ([CN]^−^) or contained the sole carbon source of glucose (Gluc), trehalose (Treh), lactose (Lact), maltose (Malt), fructose (Fruct), mannitol (Mann), sorbitol (Sorb), glycerin (Glyc), ethanol (Ethn), olive oil (OlivO), oleic acid (OleicA), or acetate (NaAc), or the sole nitrogen source of NH_4_Cl, NH_4_NO_3_, NaNO_2_, or one of 20 amino acids. All colonies were initiated by attaching 5-mm-diameter hyphal mass discs on the plates. The asterisk-marked bar for a deletion mutant differs significantly (Tukey’s HSD, *P < *0.05) from the bars unmarked for corresponding control strains in each bar group. Error bars, SD of the mean from three replicates. Download FIG S3, JPG file, 2.0 MB. Copyright © 2019 Zhang et al. 2019 Zhang et al. This content is distributed under the terms of the Creative Commons Attribution 4.0 International license.

10.1128/mSystems.00140-19.4 TABLE S1 Paired primers used for targeted gene manipulation in B. bassiana. Download Table S1, DOCX file, 0.02 MB. Copyright © 2019 Zhang et al. 2019 Zhang et al. This content is distributed under the terms of the Creative Commons Attribution 4.0 International license.

**FIG 2:**
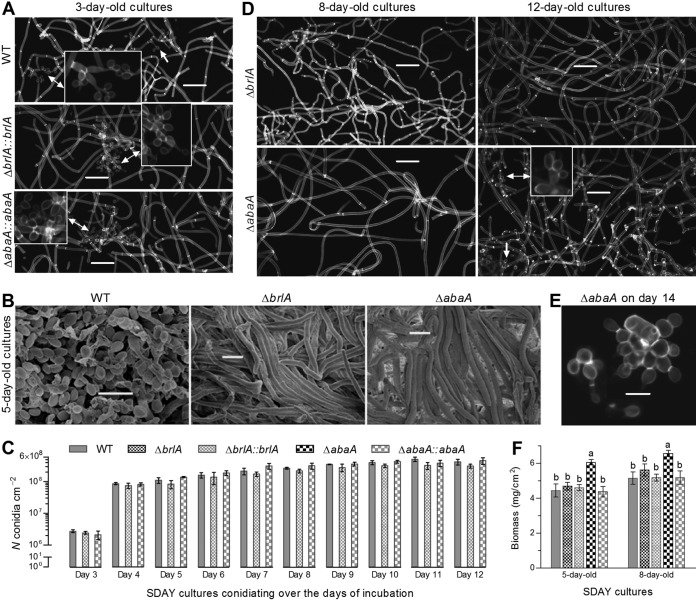
Indispensable roles of BrlA and AbaA in aerial conidiation of B. bassiana. (A) Microscopic images for initial conidiation status of three control strains in 3-day-old SDAY cultures. Double-headed arrows indicate enlarged clusters of conidiating structure (zigzag rachises) and conidia. (B) SEM images for the conidiation state of WT, Δ*brlA*, and Δ*abaA* strains in 5-day-old SDAY cultures. Note the presence of abundant conidia in the WT and of undifferentiated hyphae in the Δ*brlA* and Δ*abaA* strains. (C) Conidial yields quantified from the SDAY cultures of all tested strains during a 12-day incubation. (D) Microscopic images for the conidiation state of Δ*brlA* and Δ*abaA* strains in 8- and 12-day-old SDAY cultures. Note the absence of conidiating structures in the Δ*brlA* strain and the presence of a few seemingly conidiating cell clusters (arrowed) in the 12-day-old Δ*abaA* culture. (E) Microscopic image for the cell clusters of the 14-day-old Δ*abaA* culture, which were unable to be scattered for a suspension by supersonic vibration. (F) Biomass levels quantified from the 5- and 8-day-old SDAY cultures of two deletion mutants and control strains. All cultures were initiated by spreading 100-μl aliquots of a hyphal suspension on SDAY plates and incubated under the optimal regime of 25°C and L:D 12:12. Bars, 20 (A and D) or 5 (B and E) μm. Culture samples in panels A, D, and E were stained with calcofluor white, visualized via LSCM, and shown in black and white for clarity. Error bars, SD of the mean from three independent cultures. Different lowercase letters marked on the bars of each group denote a significant difference (Tukey’s HSD, *P* < 0.05).

Intriguingly, either *brlA* or *abaA* deletion abolished aerial conidiation during 12 days of incubation under the optimal regime on the SDAY plates spread with 100-μl aliquots of a hyphal cell suspension for culture initiation. The control strains started aerial conidiation on day 3 by formation of clustered zigzag rachises (conidiophores) and conidia (Fig. 2A), produced plenty of conidia on day 5 (Fig. 2B), and reached a peak yield of ∼5 × 10^8^ conidia cm^−2^ plate culture on day 7 or 8 (Fig. 2C). In contrast, the hyphae of Δ*brlA* and Δ*abaA* mutants were not normally differentiated during the period of optimal incubation, as revealed by scanning electronic microscopic (SEM) analysis of 5-day-old cultures (Fig. 2B) or microscopic examination of 8- or 12-day-old cultures (Fig. 2D). Interestingly, cell clusters like conidiating structures were sporadically present in the 12-day-old Δ*abaA* cultures. However, such cell clusters did not comprise the same zigzag rachises as appeared in the cultures of the control strains; the clustered cells were unable to be scattered for suspension preparation via supersonic vibration and hence were unlikely conidia (Fig. 2E). In addition, biomass levels quantified from the 5- and 8-day-old cultures of the Δ*abaA* strain were significantly higher (Tukey’s HSD, *P* < 0.05) than those from the cultures of the control strains but unaffected in the Δ*brlA* strain (Fig. 2F). The completely abolished conidiation in the absence of *brlA* or *abaA* highlights an indispensability of either BrlA or AbaA for aerial conidiation of B. bassiana.

### BrlA and AbaA are indispensable for dimorphic transition *in vitro*.

**FIG 3:**
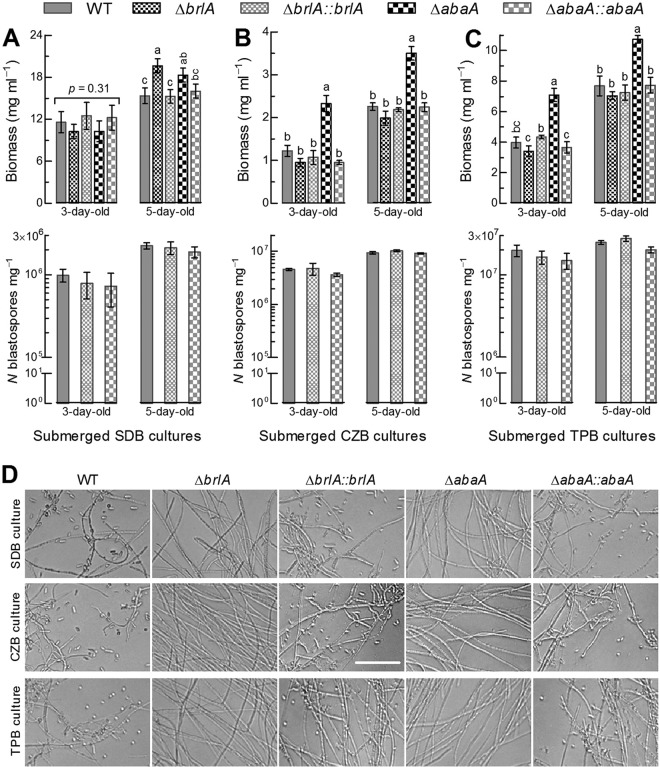
Indispensable roles of BrlA and AbaA in dimorphic transition *in vitro* of B. bassiana. (A to C) Biomass levels and blastospore yields quantified from the 3- and 5-day-old submerged cultures of two deletion mutants and their control strains in SDB, CZB, and TPB, respectively. Each culture was initiated with 1 mg fresh hyphae ml^−1^ SDB, CZB, or TPB mimicking insect hemolymph. Error bars, SD of the mean from three independent cultures. Different lowercase letters marked on the bars of each group denote a significant difference (Tukey’s HSD, *P* < 0.05). (D) Microscopic images (bar, 50 μm) for the status of blastospore production in the 3-day-old SDB, CZB, and TPB cultures of all tested strains. Note that blastospore production occurred in the control strains but was completely abolished in the Δ*brlA* and Δ*abaA* strains.

The submerged cultures of two deletion mutants and their control strains were initiated with a hyphal cell suspension (1 mg fresh biomass ml^−1^) in rich SDB (i.e., agar-free SDAY), minimal Czapek broth (CZB), and trehalose-peptone broth (TPB) mimicking insect hemolymph, followed by a 5-day incubation on a shaking bed (150 rpm) at 25°C. For each strain, mean biomass level quantified for three independent cultures at the ends of 3- and 5-day incubations was maximal in SDB (Fig. 3A), minimal in CZB (Fig. 3B), and intermediate in TPB (Fig. 3C). The Δ*abaA* mutant produced significantly more biomass than the control strains in all submerged cultures except the 3-day-old SDB culture while the Δ*brlA* mutant had biomass levels similar to those of the control strains in all cultures except the 5-day-old SDB culture. As an index of dimorphic transition *in vitro*, mean (±SD) yields at the scale of 10^6^ blastospores mg^−1^ biomass in the SDB, CZB, and TPB cultures of three control strains increased to 2.08 (±0.18), 9.64 (±0.63), and 24.20 (±3.77) on day 5 from 0.84 (±0.14), 4.32 (±0.62), and 17.09 (±2.46) on day 3, respectively. Apparently, their dimorphic transition rates in the TPB cultures were much higher than those in CZB or SDB. However, blastospore formation was completely abolished in all submerged cultures of both Δ*brlA* and Δ*abaA* mutants although their biomass levels were equal to or higher than those of the control strains. In microscopic examination, no blastospore was found in the culture samples of either deletion mutant, contrasting with the presence of abundant blastospores in the cultures of the control strains (Fig. 3D). These data indicate for the first time an absolute indispensability of BrlA or AbaA for the dimorphic transition *in vitro* of B. bassiana.

### BrlA and AbaA are indispensable for pathogenicity and dimorphic transition *in vivo*.

**FIG 4:**
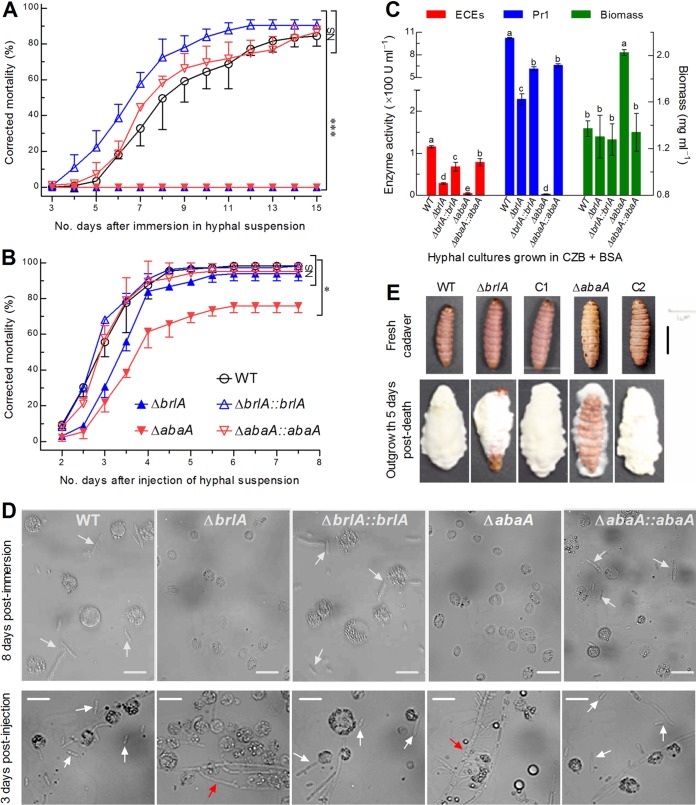
Indispensability of BrlA and AbaA for hyphal pathogenicity and dimorphic transition *in vivo* in B. bassiana. (A and B) Time-mortality trends of G. mellonella larvae immersed in a suspension of 10 mg fresh hyphae ml^−1^ for normal cuticle infection or injected with a suspension of 2 mg fresh hyphae ml^−1^ for cuticle-bypassing infection. Note that no mortality was attributed to the Δ*brlA* or Δ*abaA* strain, in contrast with high mortalities caused by three control strains (NS, not significantly different; *, *P* < 0.01; ***, *P* < 0.0001) through cuticle penetration. (C) Dry biomass of hyphal cells (right *y* axis) and total activities (U ml^−1^ culture supernatant) of extracellular enzymes (ECEs) and Pr1 proteases (left *y* axis) quantified from 60-h-old cultures in CZB, which contained 0.3% bovine serum albumin (BSA) as the sole nitrogen source for enzyme induction and fresh hyphal cells (1 mg ml^−1^) for culture initiation. Different lowercase letters marked on the bars of each group denote a significant difference (Tukey’s HSD, *P* < 0.05). Error bars, SD of the mean from three independent cultures (A to C). (D) Microscopic images (scale, 20 μm) for hyphal bodies (white arrows), hyphae (red arrows), and insect hemocytes (spherical or subspherical cells) in hemolymph samples of the larvae surviving on day 8 after immersion or day 3 after injection. (E) Images (scale, 10 mm) for fungal outgrowths (C1 and C2, complementary strains) on the surfaces of cadavers 5 days after death from injection.

Since the two deletion mutants produced neither aerial conidia nor submerged blastospores for topical application or intrahemocoel injection in standardized bioassays as described previously (6, 8), a blastospore-removed hyphal suspension (10 mg fresh biomass ml^−1^) of each control strain or deletion mutant was topically applied for normal infection through cuticular penetration by immersing three cohorts of ∼35 Galleria mellonella larvae in 30-ml aliquots of each suspension for 10 s. The infection resulted in a mean corrected mortality of 87% (±3.7%) for the control strains against the model insect (Fig. 4A). In contrast, no mortality was attributed to the topical application of the Δ*brlA* or Δ*abaA* hyphae. Moreover, 5 μl of a hyphal suspension (2 mg fresh biomass ml^−1^) was injected into the hemocoel of each larva in each of three cohorts for cuticle-bypassing infection. As a result, the injected larvae were mummified much more rapidly than those that were immersed (Fig. 4B). The injection resulted in an overall mean mortality of 97% (±3.1%) for the control strains, followed by 93% (±3.9%) attributed to the Δ*brlA* mutant and 75% (±3.6%) attributed to the Δ*abaA* mutant. Apparently, either Δ*brlA* or Δ*abaA* strain hyphae lost all ability to infect the larvae through the normal route of cuticular penetration, indicating an indispensability of BrlA or AbaA for the insect pathogenicity of *B.* bassiana. The injected mutant hyphae caused lower mortality or slower death, hinting at a possibility that certain virulence-related cellular processes could be retarded in the absence of *brlA* or *abaA*.

To gain an insight into the abolished cuticle infection, we assessed total activities of extracellular (proteolytic, chitinolytic, and lipolytic) enzymes and Pr1 proteases secreted from the 60-h-old liquid cultures grown in CZB containing 0.3% bovine serum albumin (BSA) as the sole nitrogen source for enzyme induction and initiated with fresh hyphal cells at the fixed rate of 1 mg ml^−1^. Such enzymes are likely involved in cuticle degradation essential for host infection (2). As illustrated in Fig. 4C, the activities of extracellular enzymes and Pr1 proteases quantified from the supernatants of the cultures were reduced by 75% and 78% in the Δ*brlA* mutant and 96% and 99% in the Δ*abaA* mutant, respectively, in comparison to the activities in WT. However, the mean biomass level in the CZB-BSA cultures was unaffected in the Δ*brlA* mutant and even enhanced by 47% in the Δ*abaA* mutant relative to WT. All of these changes were restored by targeted gene complementation. The results imply that the abolition of the cuticle infection could be due to blocked secretion and/or synthesis of cuticle-degrading enzymes in the absence of *brlA* or *abaA*.

To explore a possible cause for the delayed lethal action of the injected mutant hyphae, we examined the status of dimorphic transition *in vivo* through microscopic examination of the hemolymph samples taken from the larvae surviving after topical application or intrahemocoel injection as reported previously (4, 6). On day 8 after topical application, the control strains formed abundant hyphal bodies in host hemolymph whereas either the Δ*brlA* or Δ*abaA* mutant formed no hyphal body in the host hemolymph comprising many more intact host hemocytes (upper panels in Fig. 4D). On day 3 after injection, the hyphae of the control strains also formed abundant hyphal bodies in the host hemolymph, but discrete hyphal bodies were rarely found in the host hemolymph injected with the mutant hyphae, which seemingly grew *in vivo* by direct extension (lower panels in Fig. 4D). Intriguingly, the injected Δ*abaA* strain hyphae became thicker in the host hemolymph and formed many short branches nearly perpendicular to the stem, implying slower extension of the branched Δ*abaA* strain hyphae than of the unbranched Δ*brlA* strain hyphae in the host hemocoel. In addition, the control strains formed a heavy layer of outgrowth on the surfaces of all cadavers 5 days after death from the injection (Fig. 4E). In contrast, the fungal outgrowth was thin for the Δ*brlA* mutant and very sparse for the Δ*abaA* mutant, leaving some or most of the cadaver surfaces baldly exposed. These observations demonstrated an inability of the two deletion mutants to form hyphal bodies *in vivo* and hence an indispensability of either BrlA or AbaA for dimorphic transition in the host hemocoel. The slower host death from the injected mutant hyphae was due to mummification by hyphal extension or branching rather than yeast-like budding, which accelerates fungal proliferation in the host hemocoel and hence host mummification to death (4, 6).

### Regulatory roles of BrlA and AbaA in global gene expression.

The roles of BrlA and AbaA acting as transcription factors in the central pathway of B. bassiana were examined by constructing and analyzing the transcriptomes of Δ*brlA*, Δ*abaA*, and WT strains based on the cDNAs derived from two 84-h-old SDAY cultures (replicates), which were grown under the optimal regime. Conidiation was rapidly developing in the WT cultures prepared. The replicated transcriptomes showed a high correlation (*R*^2^ = 0.993 for WT, 0.971 for Δ*brlA* strain, and 0.986 for Δ*abaA* strain), indicating a desired repeatability. The resultant transcriptomes comprised almost all annotated genes (10,363) and hundreds of genes not annotated in the fungal genome (37).

**FIG 5:**
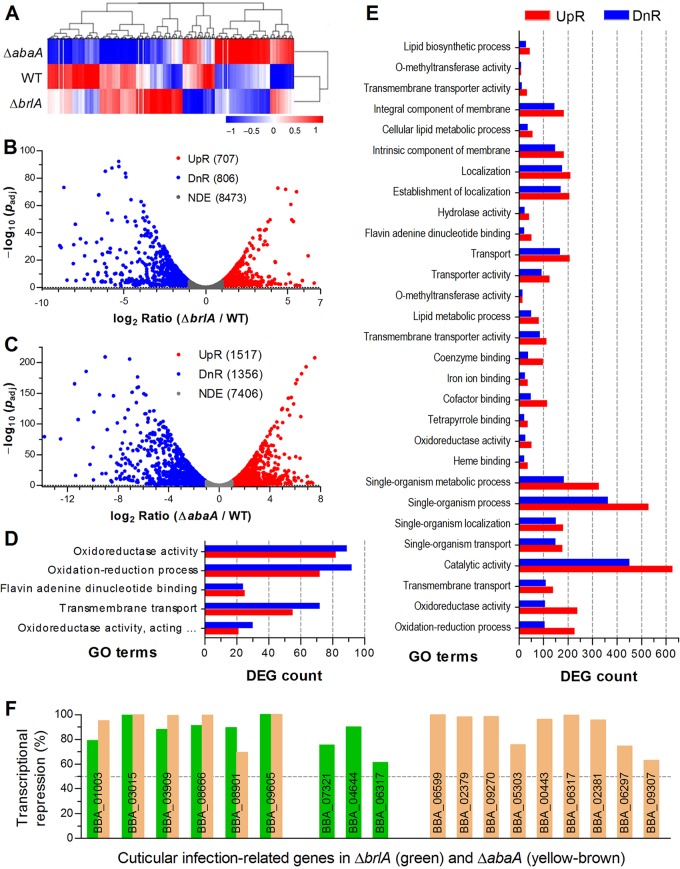
Regulatory roles of BrlA and AbaA in global gene expression of B. bassiana. (A) Cluster analysis of differentially expressed genes (DEGs) in the transcriptomes based on the 84-h-old SDAY cultures of Δ*brlA*, Δ*abaA*, and WT strains grown under the optimal regime of 25°C and L:D 12:12. (B and C) Distributions of log_2_ ratios (≥1 or ≤−1) and adjusted *P* values (*P*_adj_ < 0.05) for 1,513 and 2,869 DEGs identified from Δ*brlA* and Δ*abaA* strains, respectively. (D and E) Significantly enriched GO terms (corrected *P* < 0.05) for the DEGs of Δ*brlA* and Δ*abaA* strains, respectively. (F) Transcriptional repression levels (calculated from antilogarithm of log_2_ ratio) of cuticle infection-related genes found in the transcriptomes of Δ*brlA* and Δ*abaA* strains relative to WT.

Compared to the WT strain, the Δ*brlA* mutant had 707 and 806 genes significantly upregulated (log_2_ ratio, 1.00 to 6.62) and downregulated (log_2_ ratio, −8.92 to −1.00), respectively (Fig. 5B; see also Table S2). Significantly upregulated (log_2_ ratio, 1.00 to 7.56) and downregulated (log_2_ ratio, −13.80 to −1.00) genes increased to 1,513 and 1,356 in the Δ*abaA* strain, respectively (Fig. 5C; also Table S3). These differentially expressed genes (DEGs) took up 14.6% of the whole genome in the Δ*brlA* strain while the proportion surprisingly increased to 27.7% in the Δ*abaA* strain. Many more genes differentially expressed in the Δ*abaA* than in the Δ*brlA* mutant could be likely due to the increasing role of AbaA at the rapidly developing stage of conidiation, since *brlA* was transcriptionally activated earlier than *abaA* (Fig. 1A) for initiation of conidiation (Fig. 3A to C). Intriguingly, the top 10% of the repressed genes (log_2_ ratio, ≤−3.2) in the Δ*brlA* (150) and Δ*abaA* (350) strains contained the same proportion (40%) encoding hypothetical proteins.

10.1128/mSystems.00140-19.5 TABLE S2 List of DEGs identified in Δ*brlA* mutant versus wild-type strain of B. bassiana. Download Table S2, XLSX file, 0.1 MB. Copyright © 2019 Zhang et al. 2019 Zhang et al. This content is distributed under the terms of the Creative Commons Attribution 4.0 International license.

10.1128/mSystems.00140-19.6 TABLE S3 List of DEGs identified in Δ*abaA* mutant versus wild-type strain of B. bassiana. Download Table S3, XLSX file, 0.2 MB. Copyright © 2019 Zhang et al. 2019 Zhang et al. This content is distributed under the terms of the Creative Commons Attribution 4.0 International license.

Revealed by gene ontology (GO) analysis, 562 Δ*brlA* DEGs (255 up- and 307 downregulated) were enriched to five GO terms (Fig. 5D; see also Table S4). Three of the GO terms were involved in molecular function (oxidoreductase activity, acting on paired donors, with incorporation or reduction of molecular oxygen; flavin adenine dinucleotide binding; and oxidoreductase activity), and two were involved in biological processes (oxidation-reduction process and transmembrane transport), but no term fell into the cellular component. Since many genes may function in multiple cellular processes and events, 7,343 Δ*abaA* DEGs (4,367 up- and 2,976 downregulated) were significantly enriched into 29 GO terms covering a much wider array of molecular functions (16) and biological processes (12) as well as cellular components (1), as shown in Fig. 5E and Table S5. These analyses hint at a more critical role of AbaA than of BrlA in transcriptional regulation of downstream genes for rapid development of the fungal conidiation activated earlier by BrlA. In addition, 62 and 343 DEGs of Δ*brlA* and Δ*abaA* strains (Tables S6 and S7) were enriched into 8 and 12 KEGG pathways, respectively, at the level of *P < *0.05. However, the corrected *P* values (0.27 to 0.52) indicated an insignificance for all KEGG enrichments except that for the degradation of aromatic compounds in the Δ*abaA* strain (corrected *P = *0.039).

10.1128/mSystems.00140-19.7 TABLE S4 Lists of enriched GO terms and DEGs in Δ*brlA* mutant versus wild-type strain of B. bassiana. Download Table S4, XLSX file, 0.04 MB. Copyright © 2019 Zhang et al. 2019 Zhang et al. This content is distributed under the terms of the Creative Commons Attribution 4.0 International license.

10.1128/mSystems.00140-19.8 TABLE S5 Lists of enriched GO terms and DEGs in Δ*abaA* mutant versus wild-type strain of B. bassiana. Download Table S5, XLSX file, 0.5 MB. Copyright © 2019 Zhang et al. 2019 Zhang et al. This content is distributed under the terms of the Creative Commons Attribution 4.0 International license.

10.1128/mSystems.00140-19.9 TABLE S6 Lists of enriched KEGG pathways and DEGs in Δ*brlA* mutant versus wild-type strain of B. bassiana. Download Table S6, XLSX file, 0.01 MB. Copyright © 2019 Zhang et al. 2019 Zhang et al. This content is distributed under the terms of the Creative Commons Attribution 4.0 International license.

10.1128/mSystems.00140-19.10 TABLE S7 Lists of enriched KEGG pathways and DEGs in Δ*abaA* mutant versus wild-type strain of B. bassiana. Download Table S7, XLSX file, 0.04 MB. Copyright © 2019 Zhang et al. 2019 Zhang et al. This content is distributed under the terms of the Creative Commons Attribution 4.0 International license.

### BrlA and AbaA regulate expression of multiple genes essential for cuticle infection but nonessential for the dimorphic switch.

The transcriptomes were further analyzed for an insight into why the hyphae of either the Δ*brlA* or Δ*abaA* mutant lost all ability to infect the insect through cuticle penetration. Nine and 15 genes likely involved in host adhesion and cuticle degradation were sharply repressed in Δ*brlA* and Δ*abaA* strains (Fig. 5F), respectively. Among those, six genes were simultaneously repressed in both deletion mutants, including the class I hydrophobin gene (BBA_03015), critical for cell hydrophobicity and pathogenicity (38); the filamentous hemagglutinin/adhesin gene (BBA_03909), required for host adhesion and infection (39); and three genes encoding thermostable alkaline protease (peptidase S8/S53, subtilisin/kexin/sedolisin; BBA_08901) acting as a virulence factor (40), a subtilase-like protein (BBA_01003), and a chitinase-like protein (BBA_09605) involved in cuticle degradation. Three other genes repressed in the Δ*brlA* strain encode two proteases (BBA_07321 and BBA_04644) and one chitinase-like protein (BBA_06317). Nine other genes repressed in the Δ*abaA* strain encode another hydrophobin-like protein (BBA_06599), one adhesin (BBA_02379), three proteases or subtilase-like proteins (BBA_09270, BBA_05303, and BBA_00443), and four chitinases or chitinase-like proteins (BBA_06317, BBA_02381, BBA_06297, and BBA_09307). The activities of such proteins or enzymes are considered to be very important for the success of fungal infection through cuticle penetration (2). Transcriptional repressions of these putative cuticle-degrading enzyme genes in Δ*brlA* and Δ*abaA* strains correlated well with markedly reduced activities of extracellular enzymes and Pr1 proteases, suggesting essential roles of both BrlA and AbaA in their transcription regulation and hence in the normal infection of B. bassiana through cuticular penetration.

However, almost all of the homologous genes that are considered to control the dimorphic switch in plant and human mycopathogens (18) were not up- or downregulated in the Δ*brlA* or Δ*abaA* mutant at the significant levels of log_2_ ratios of ≥1 or ≤−1 and of adjusted *P* of <0.05 (Table 2). Instead, two other central pathway genes and downstream *vosA* were more consistently repressed than those putatively acting in the dimorphic switch of the Δ*brlA* or Δ*abaA* strain, such as *drkA*, *cdc42*, *pakA*/*B*, *racA*, and *ras1*/*2*, which presumably function in two-component, G protein, Ras, and cAMP signaling systems. Neither were almost all of the genes encoding MAPK-cascaded components differentially expressed at significant levels. These data implied that BrlA and AbaA served as master regulators of both aerial conidiation and submerged blastospore production (dimorphic transition) while the signaling systems other than the central pathway had little role in shutting down the dimorphic switch of the Δ*brlA* or Δ*abaA* strain. In other words, it is either BrlA or AbaA that governs the dimorphic switch *in vitro* and *in vivo* in B. bassiana.

**TABLE 2:** Dimorphic transition-associated genes found in Δ*brlA* and Δ*abaA* transcriptomes

Gene category and name^*a*^	Tag locus^*b*^	Annotation	Δ*brlA*/WT		Δ*abaA*/WT	
			log_2_ *R*	*P*_adjusted_	log_2_ *R*	*P*_adjusted_
Central developmental pathway						
*brlA*	BBA_07544	C_2_H_2_ transcription factor BrlA			−6.215	0.0000
*abaA*	BBA_00300	Conidiation factor AbaA	−1.991	0.0000		
*wetA*	BBA_06126	Conidial maturation factor WetA	−0.730	0.0065	−0.794	0.0008
*vosA*	BBA_01023	Velvet protein VosA	−0.500	0.0815	−1.851	0.0000
Putative dimorphic switch genes						
*cdc42*	BBA_01874	Cell division control protein 42	0.315	0.3189	−0.334	0.1225
*drkA*	BBA_01218	Group III histidine kinase HK3	0.568	0.0299	−0.244	0.2699
*pakA*	BBA_07438	PAK kinase	−0.247	0.4557	−0.442	0.0418
*pakB*	BBA_04136	Protein kinase CHM1	0.110	0.7880	−0.309	0.1519
*racA*	BBA_08808	Small GTPase, Rho type	0.342	0.2468	0.133	0.5968
*ras1*	BBA_04387	Ras GTPase 1 (Ras1)	−0.274	0.4143	−0.235	0.3013
*ras2*	BBA_04671	Ras GTPase 2 (Ras2)	−0.311	0.5188	−0.441	0.0713
*ryp1*	BBA_06411	Protein Ryp1	0.142	0.9702	−1.126	0.0004
*ryp2*	BBA_05501	Protein Ryp2	0.462	0.1065	0.226	0.3463
MAPK cascaded pathways						
*ste11*	BBA_02280	MAPK kinase kinase Ste11	0.835	0.0011	0.821	0.0040
*ste7*	BBA_04254	MAPK kinase Ste7	−0.355	0.2180	−0.543	0.0077
*fus3*	BBA_01244	MAPK Fus3	−0.250	0.4698	−0.101	0.6658
*ssk2*	BBA_00937	MAPK kinase kinase Ssk2	0.027	0.9620	0.076	0.7790
*pbs2*	BBA_02330	MAPK kinase Pbns2	0.050	0.9158	−0.026	0.9326
*hog1*	BBA_05209	MAPK Hog1	0.814	0.0298	1.207	0.0240
*bck1*	BBA_01318	MAPK kinase kinase Bck1	−0.065	0.8808	−0.300	0.1701
*mkk1*	BBA_01095	MAPK kinase Mkk1	−0.109	0.7935	−0.135	0.5861
*slt2*	BBA_03334	MAPK Slt2	−1.079	0.0000	−0.481	0.0169

a Putative dimorphic switch genes are homologous to those of human mycopathogens listed in a review (18).

b Gene accession codes in B. bassiana genome under the NCBI accession no. NZ_ADAH00000000.

## DISCUSSION

BrlA and AbaA are evidently localized in both the cytoplasm and nucleus and sequentially activated at the transcription level in B. bassiana like their orthologs in the aspergilli (21, 26). Our results demonstrate essential roles of both developmental activators in the asexual cycle *in vitro* and *in vivo* of a filamentous fungal insect pathogen, as discussed below.

In B. bassiana, submerged blastospore production *in vitro*, particularly in the TPB mimic of insect hemolymph, often correlates with dimorphic transition *in vivo*, virulence, and activated genes in the central developmental pathway. For instance, transcriptional repression of *brlA* or *abaA* by ≥90% (Table 1) resulted in severe or extremely severe defects in blastospore production, blocked or inhibited dimorphic transition *in vivo*, and greatly attenuated virulence or abolished host infection in the absence of *gcn5*, *rpd3*, *vvd*, or *vlp4* (6, 8, 11, 14). In this study, hyphal pathogenicity to insect and blastospore production *in vitro* and *in vivo* was completely abolished due to the deletion of *brlA* or *abaA*. Previously, normal cuticle infection was also abolished in Δ*fus3*, Δ*ste7*, and Δ*ste11* mutants of B. bassiana due to reduced activities of cuticle-degrading enzymes largely attributed to their severe growth defects on oligotrophic substrata (12, 41, 42). However, the three genes in the Fus3 cascade were not differentially expressed in the transcriptomes of Δ*brlA* and Δ*abaA* mutants. Neither was hyphal growth of either mutant retarded in various media. The inability for the hyphae of the Δ*brlA* or Δ*abaA* strain to infect the insect through cuticular penetration could be attributable to the markedly reduced transcripts of multiple genes critical for host adhesion and cuticle degradation. This inference is supported by sharply reduced activities of extracellular enzymes and Pr1 proteases in both deletion mutants. Thus, the present and previous studies indicate an indispensability of either BrlA or AbaA for insect pathogenicity of B. bassiana and also unveil a novel molecular mechanism distinct from the control of the dimorphic switch by the signaling systems other than the central pathway in plant and human mycopathogens (18). Previously, only AbaA was revealed to be involved in the dimorphic switch of *Talaromyces* (formerly *Penicillium*) *marneffei* (43). We consider that the distinct mechanisms underlying the dimorphic switch of these fungal pathogens may stem from distinct host-pathogen interactions and their peculiar lifestyles. The dimorphic switch of an insect mycopathogen occurs only after the success of hyphal infection through cuticular penetration and is required for acceleration of intrahemocoel fungal propagation by yeast-like budding and of host mummification to death. In contrast, dimorphic plant or human mycopathogens require a switch from the usual yeast growth to filamentous growth for initiation of host infection by penetration through host tissues (34, 36). Our inference is supported by abolished dimorphic transition *in vitro* and *in vivo* and almost all signaling genes not being differentially expressed in either the Δ*brlA* or the Δ*abaA* strain but being considered to control the dimorphic switch in plant and human mycopathogens (18). Thus, both BrlA and AbaA serve as master regulators of the dimorphic switch in B. bassiana.

Moreover, the block of the central development pathway by the *brlA* or *abaA* deletion also led to abolishment of aerial conidiation but had no negative impact on hyphal growth in various agar or broth media. During a prolonged 12-day period of optimal incubation for conidiation on the standard medium, no sign of hyphal differentiation was observed in the Δ*brlA* mutant while the Δ*abaA* mutant displayed sporadic cell clusters at the end of incubation. However, the clustered cells failed to form short rachises for support of conidial production and hence were not the normal spore balls that comprise many zigzag rachises and conidia and can be readily scattered for a uniform suspension. We speculate that the strange cell clusters could likely result from abnormal hyphal differentiation and hence are similar to aberrant and nonfunctional phialides observed in Penicillium digitatum Δ*abaA* (44). Thus, the abolished conidiation of Δ*brlA* and Δ*abaA* mutants in this study is consistent with previous observations from the same mutants of other filamentous fungi (26, 43, 44). This reinforces highly conserved roles of both BrlA and AbaA in the regulation of asexual development and life cycle in filamentous fungi.

Finally, our transcriptomic analyses revealed that the deletion of *brlA* or *abaA* resulted in similar repression of the downstream gene *wetA*, which has been shown to regulate conidial maturation and cell wall integrity in the central pathway of B. bassiana (32). In the previous study, knockout mutation of *wetA* led to 98% reduction in conidial yield and much more attenuated virulence via intrahemocoel injection than via normal cuticle infection while the fungal virulence through either mode of infection was slightly or insignificantly affected when the downstream *vosA* lost function. This hints at a possible role of WetA instead of VosA in the dimorphic switch of B. bassiana required for yeast-like budding propagation to accelerate host mummification. However, it is impossible for the limited repression level of *wetA* to completely shut down the dimorphic switch *in vitro* and *in vivo* in Δ*brlA* and Δ*abaA* strains. Like aerial conidiation, therefore, submerged dimorphic transition *in vitro* and *in vivo* is a process of asexual development that is governed by BrlA and AbaA in the central pathway of B. bassiana.

## MATERIALS AND METHODS

### Bioinformatic analysis of BrlA and AbaA in B. bassiana.

The A. nidulans BrlA (EAA66002) and AbaA (EAA35779) sequences were used as queries to search through the B. bassiana genome (37) under NCBI accession no. NZ_ADAH00000000 via online BLASTp analysis at https://blast.ncbi.nlm.nih.gov/Blast.cgi. The sequences of BrlA and AbaA orthologs found in the fungal genome were aligned for structural comparison with the queries and counterparts of other filamentous, yeast, or yeast-like fungi using the SMART program at http://smart.embl-heidelberg.de/, followed by phylogenetic analysis with a neighbor-joining method in MEGA7 software at http://www.megasoftware.net/.

### Transcriptional profiling of *brlA* and *abaA*.

The WT strain was incubated for 7 days under the optimal regime of 25°C and L:D 12:12 on cellophane-overlaid SDAY (4% glucose, 1% peptone, and 1.5% agar plus 1% yeast extract) plates, which were spread with 100-μl aliquots of a 10^7^-conidia ml^−1^ suspension for culture initiation. From the end of the 24-h incubation onward, total RNAs were separately extracted daily from three plate cultures using an RNAiso Plus reagent kit (TaKaRa, Dalian, China) and reverse transcribed into cDNAs using a PrimeScript reverse transcription (RT) reagent kit (TaKaRa). Each of the cDNA samples (standardized by dilution) was used as a template to assess transcript levels of *brlA* and *abaA* via real-time quantitative PCR (qPCR) with paired primers (see Table S1 in the supplemental material) under the action of SYBR Premix Ex Taq (TaKaRa). The fungal 18S rRNA was used as an internal standard. The threshold cycle (2^−ΔΔ^*^CT^*) method (45) was used to compute the relative transcript level of *brlA* or *abaA* in the WT strain on a given day with respect to the standard level at the end of the 24-h incubation.

### Subcellular localization of BrlA and AbaA.

Transgenic strains strongly expressing BrlA::GFP and AbaA::GFP fusion proteins in the WT strain, respectively, were created as described previously (6). Each transgenic strain was incubated for full conidiation on SDAY. The resultant conidia were suspended in SDB and incubated on a shaking (150-rpm) bed for 3 days at 25°C in the L:D cycles of 24:0, 12:12, and 0:24. Hyphal cells taken from the cultures of each strain in each L:D cycle were stained with the nucleus-specific dye DAPI (4′,6′-diamidine-2′-phenylindole dihydrochloride; Sigma) and visualized. LSCM images for BrlA::GFP and AbaA::GFP fusion proteins expressed in the stained cells were merged with an image browser to judge subcellular localization of each target protein in response to the L:D cycles. To verify a nuclear localization of each fusion protein, green fluorescence intensities were measured from the cytoplasm and nuclei of at least 10 hyphal cells (one nucleus per cell) using ImageJ software at https://imagej.nih.gov/ij/. Relative accumulation levels of each fusion protein in the nuclei of the hyphal cells incubated in the L:D cycles of 0:24, 12:12, and 24:0 were calculated as the ratios of nuclear to cytoplasmic fluorescence intensities.

### Generation of *brlA* and *abaA* mutants.

The genes *brlA* and *abaA* (tag loci: BBA_07544 and BBA_00300, respectively) were deleted from the WT strain as described previously for the deletion of *wetA* or *vosA* (32). Briefly, 5′ and 3′ partial coding/flanking sequences of each gene were amplified from the genomic DNA of the WT strain using paired primers (Table S1) and inserted into linearized p0380-bar at appropriate enzyme sites. The resultant plasmids p0380-5′*x*-bar -3′*x* (*x* = *brlA* or *abaA*) were integrated into the WT via *Agrobacterium*-mediated transformation for targeted gene deletion. For targeted gene complementation, the full-length coding sequence of *brlA* or *abaA* with flanking regions was cloned from the WT DNA with paired primers, digested with the appropriate restriction enzyme, and inserted into linearized p0380-sur-exchange. The resultant p0380-sur-*x* was ectopically integrated into the protoplasts of each deletion mutant via polyethylene glycol-mediated transformation (46). The used protoplasts were released from the hyphal cells collected from the 3-day-old SDB culture of each deletion mutant by suspending 100-mg aliquots of hyphal cells (fresh weight) in 2 ml of 1.2 M sorbitol containing 1% snailase and 1% lysing enzyme (Sigma) for 5-h cell wall lysing at 37°C. The released protoplasts were collected by filtration through lens-cleaning tissues, rinsed repeatedly with 1.2 M sorbitol, and used as recipients for targeted gene complementation. Putative deletion or complementary mutant colonies grown on a selective medium were screened by *bar* resistance to phosphinothricin (200 μg ml^−1^) or *sur* resistance to chlorimuron ethyl (10 μg ml^−1^) and sequentially identified through PCR and Southern blot analyses with paired primers and amplified probes (Table S1). The positive *brlA* and *abaA* mutants with expected recombinant events verified (Fig. S2) were evaluated in parallel with the WT strain in the following experiments comprising three independent cultures or samples taken from the cultures.

### Assessments of growth rates on different media.

Hyphal mass plugs (5-mm diameter) were bored from the culture of each strain grown on cellophane-overlaid SDAY (CO-SDAY) plates for 3 days at 25°C and attached centrally to the plates of SDAY, CZA (3% sucrose, 0.3% NaNO_3_, 0.1% K_2_HPO_4_, 0.05% KCl, 0.05% MgSO_4_, and 0.001% FeSO_4_ plus 1.5% agar), and amended CZAs containing different carbon or nitrogen sources. The amended CZAs were prepared by deleting 3% sucrose, 0.3% NaNO_3_, or both from CZA; replacing the sole carbon source with 3% glucose, trehalose, lactose, fructose, maltose, mannitol, sorbitol, glycerol, ethanol, sodium acetate (NaAc), oleic acid, or olive oil; and replacing the sole nitrogen source with 0.3% NH_4_Cl, NH_4_NO_3_, NaNO_2_, or one of 20 amino acids, respectively. After a 7-day incubation at 25°C and 12:12 h, all colony diameters were measured as indices of radial growth rates using two measurements taken perpendicular to each other across the center.

### Assessment of conidiation capacity.

Three 100-μl aliquots of a hyphal suspension (fresh hyphal mass, 1 mg ml^−1^) per strain were evenly spread on SDAY plates and incubated for 12 days under the optimal regime of 25°C and L:D 12:12. From the end of the 3-day incubation onward, three plugs (5-mm diameter) were bored daily from each plate culture using a cork borer. Each plug was placed in 1 ml of 0.02% Tween 80 for the release of its conidia by supersonic vibration. Three samples taken from each of the resultant suspensions were used for assessment of conidial concentration with a hemocytometer, followed by converting the concentration to the number of conidia produced per unit area (cm^2^) of plate culture. During the period of incubation, the conidiation state of fungal mass samples taken from the cultures of each strain was stained with calcofluor white (a dye specific to cell wall) and observed under a microscope or directly examined via SEM as described elsewhere (6).

### Assessment of dimorphic transition *in vitro*.

Hyphal cells collected from the 3-day-old SDB cultures of each strain were rinsed twice with sterile water and resuspended in 50-ml aliquots of fresh SDB, CZB (i.e., agar-free CZA), and TPB, which was a modified CZB containing the sole carbon source of 3% trehalose and the sole nitrogen source of 0.5% peptone and which mimicked insect hemolymph. Possible blastospores in each culture were removed by filtration through lens-cleaning tissues. All of the aliquots in flasks were standardized to a final concentration of fresh hyphal mass of 1 mg ml^−1^ and incubated for 5 days with shaking (150 rpm) under the optimal regime. At the end of 3- and 5-day incubations, three 50-μl samples were taken from each of three flasks per strain in each broth. The blastospore concentration (count ml^−1^ culture) was assessed from each sample using a hemocytometer. The remaining culture of each flask was dried by pumping in a vacuum, followed by estimation of biomass level (mg ml^−1^). The two quantities were used to compute the absolute blastospore yield (number of blastospores mg^−1^ biomass) as an index of dimorphic transition *in vitro* in each submerged culture.

### Assays for hyphal pathogenicity and activities of extracellular enzymes involved in cuticle degradation.

Since Δ*brlA* and Δ*abaA* strains produced neither conidia nor blastospores, the 3-day-old SDB cultures of all deletion mutants and control strains prepared as described above were filtered through lens-cleaning tissues for removal of possible blastospores. The collected hyphae of each strain were suspended in 0.02% Tween 80 and standardized to a concentration of fresh hyphae of 2 or 10 mg ml^−1^. For topical application, three cohorts (replicates) of ∼35 G. mellonella larvae were separately immersed for 10 s in 30-ml aliquots of each hyphal suspension (10 mg ml^−1^) for normal infection through cuticular penetration. Alternatively, 5 μl of each hyphal suspension (2 mg ml^−1^) was injected into the hemocoel of each larva in each cohort. The same volume of 0.02% Tween 80 was used as a control in each bioassay. All treated cohorts were maintained at 25°C and 12:12 h and monitored every 12 or 24 h for survival/mortality records until the records no longer changed for two consecutive days. The time-mortality trends of the larvae treated with each strain were corrected using the mortality records in the control. On day 8 after immersion or day 3 after injection, hemolymph samples were taken from surviving larvae and examined for the presence/absence of hyphal bodies under a microscope as described elsewhere (4, 6, 8). Images for fungal outgrowths on the surfaces of cadavers were taken after 5-day maintenance under optimal conditions.

Total activities of extracellular enzymes and Pr1 proteases involved in fungal infection through cuticular penetration were quantified from the liquid cultures of each strain as described previously (4, 6, 8). Briefly, 50-ml aliquots of a hyphal suspension (fresh hyphae, 1 mg ml^−1^) in CZB containing 0.3% BSA as the sole nitrogen source for induction of enzyme production were incubated on a shaking bed (150 rpm) for 60 h at 25°C. The cultures were centrifuged at 4°C. Hyphal biomass in each culture was quantified after vacuum drying. Total activities of extracellular enzymes and Pr1 proteases in each supernatant were assessed by reading optical densities at 442 and 410 nm (OD_442_ and OD_410_), respectively. One unit of enzyme activity was defined as an enzyme amount required for an OD_442_ or OD_410_ increase by 0.01 after a 1-h reaction of each extract relative to a blank control. Total activities were expressed as U ml^−1^ supernatant.

### Analyses of *brlA*- and *abaA*-specific transcriptomes.

Two 84-h-old cultures (replicates) of Δ*brlA*, Δ*abaA*, and WT strains grown on cellophane-overlaid SDAY plates under the optimal regime were sent to Novogene (Beijing, China) for construction and analysis of transcriptomes. Total RNAs were separately extracted from the two cultures of each strain. The mRNAs were isolated from total RNAs using magnetic oligo(dT) beads and fragmented into segments. The resultant mRNA fragments were used as the templates to synthesize the first-strand cDNAs with random hexamer primers. The second-strand cDNAs were synthesized using the first-strand cDNAs as the templates. Each of the double-strand cDNAs was purified and end repaired, and single adenines were added to the ends of the cDNA molecules. Finally, the cDNA library was constructed by adding proper adaptors to the cDNA for sequencing on an Illumina HiSeq platform.

Clean tags were generated by filtering all raw reads from the cDNA sequencing and then mapped to the B. bassiana genome (37) based on log_2_ ratio (Δ*brlA*/WT or Δ*abaA*/WT strain) of ≤−1 (downregulated) or ≥1 (upregulated) at the significance of corrected *P < *0.05. All data were normalized as fragments per kilobase of exon per million fragments mapped (FPKM). All identified DEGs were functionally annotated with known or putative gene information in the nonredundant NCBI protein databases and subjected to GO analysis (http://www.geneontology.org/) for recognition of GO terms falling into biological process, molecular function, and cellular component. Further, Kyoto Encyclopedia of Genes and Genomes (KEGG) analysis (http://www.genome.jp/kegg/) was performed to enrich the DEGs into various KEGG pathways at the significance level of *P* or corrected *P* of <0.05.

### Data availability.

All data generated or analyzed during this study are included in the published paper and associated supplemental files. All transcriptomic data aside from those reported in supplemental files (Tables S2 to S7) of this paper are available at the NCBI's Gene Expression Omnibus under the accession no. GSE132277.
