# Supplementary figures and images for: BrlA and AbaA Govern Virulence-Required Dimorphic Switch, Conidiation, and Pathogenicity in a Fungal Insect Pathogen
Source: mSystems. 2019 Jul 9;4(4):e00140-19. doi: 10.1128/mSystems.00140-19 (PMC6616149; doi:10.1128/mSystems.00140-19)

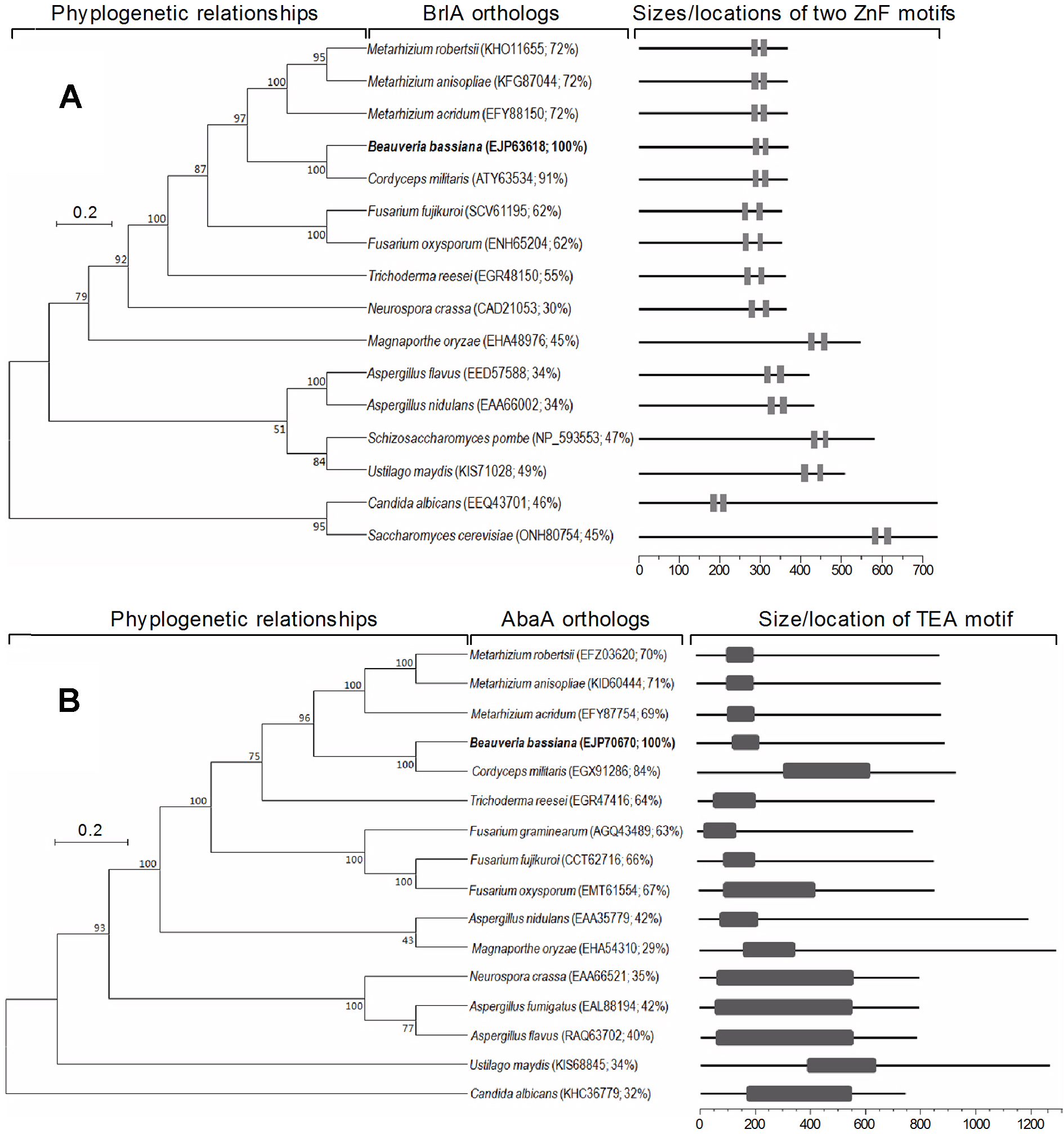

Supplement: FIG S1 [file mSystems.00140-19-sf001.jpg]

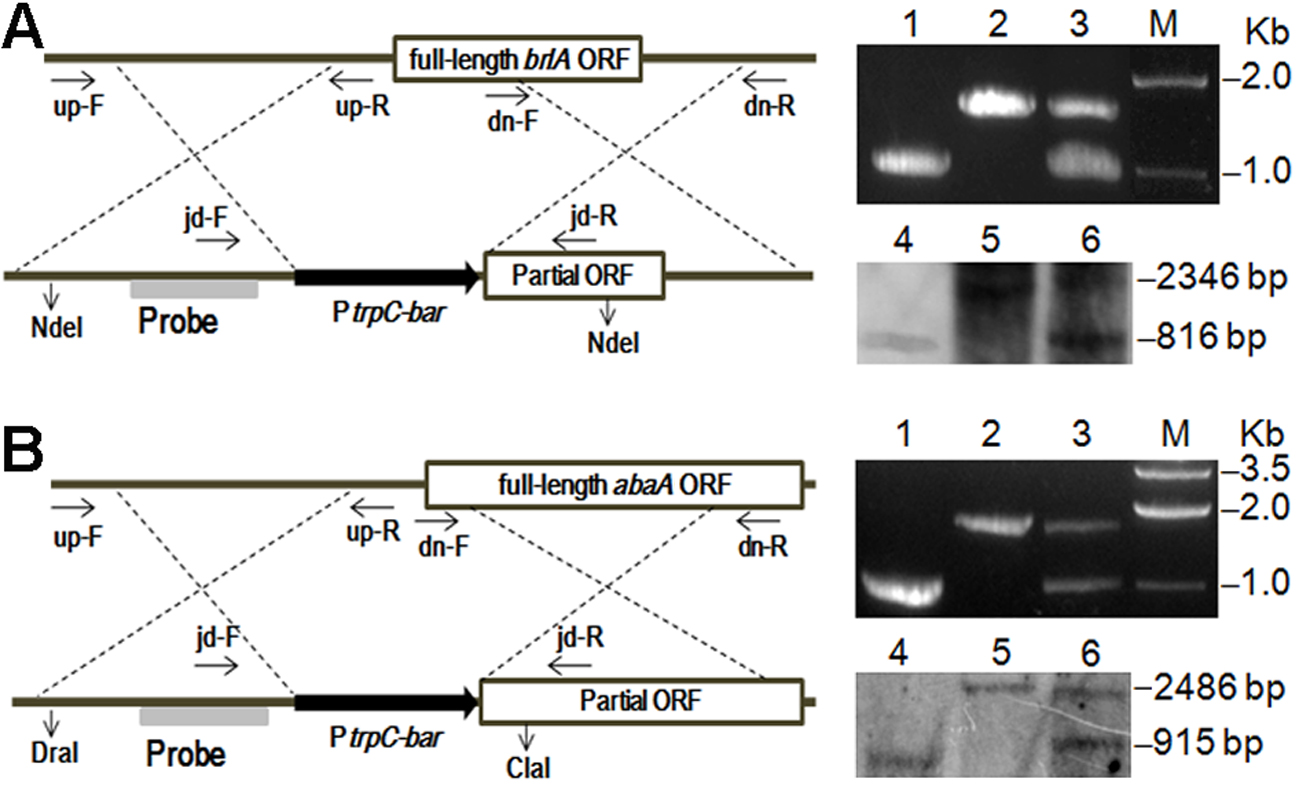

Supplement: FIG S2 [file mSystems.00140-19-sf002.jpg]

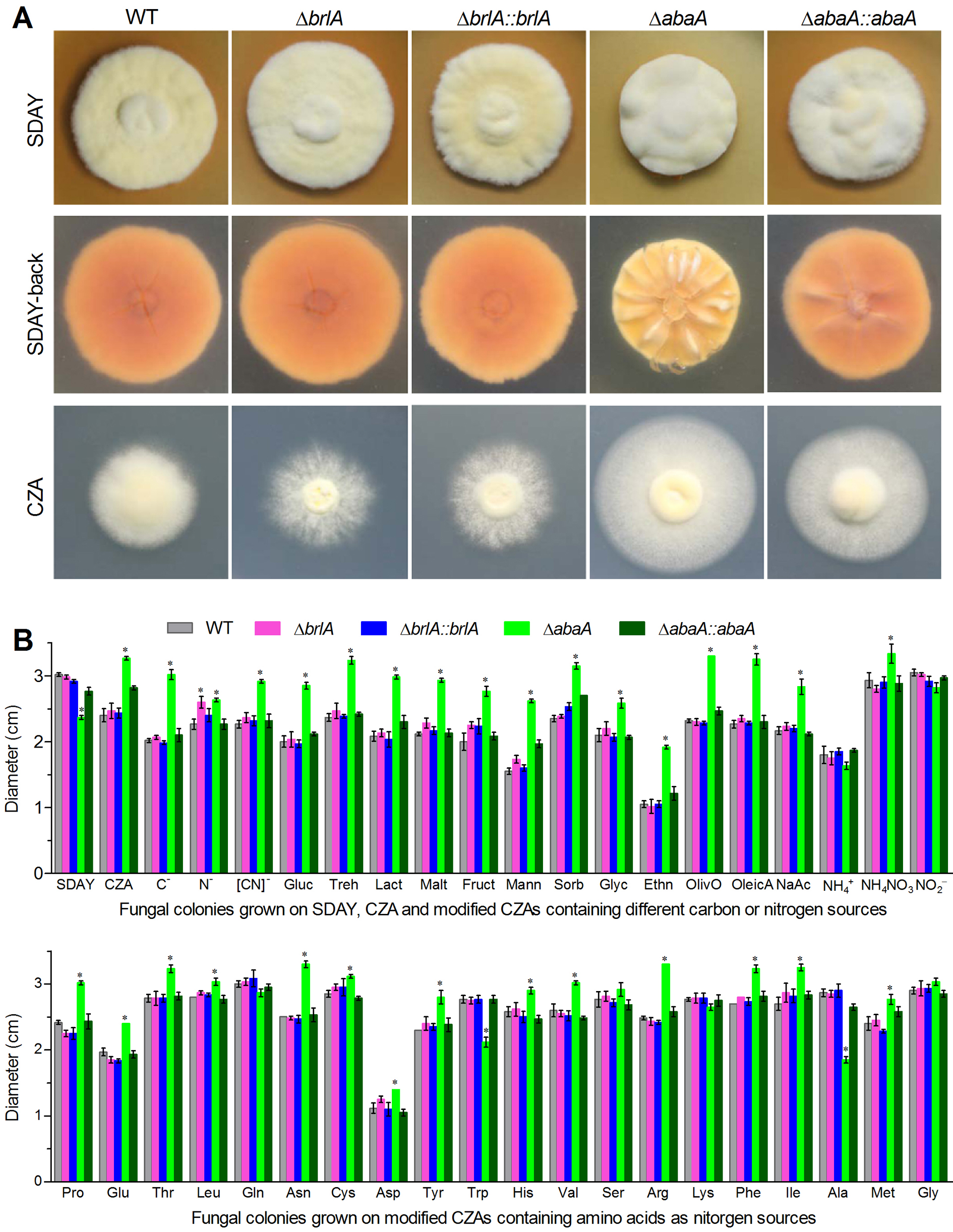

Supplement: FIG S3 [file mSystems.00140-19-sf003.jpg]
