# Supplementary material for: BrlA and AbaA Govern Virulence-Required Dimorphic Switch, Conidiation, and Pathogenicity in a Fungal Insect Pathogen
Source: mSystems. 2019 Jul 9;4(4):e00140-19. doi: 10.1128/mSystems.00140-19 (PMC6616149; doi:10.1128/mSystems.00140-19)
Supplement: TABLE S1 [file mSystems.00140-19-st001.docx]

| Primers | Paired sequences (5′−3′)* | Purpose |
| --- | --- | --- |
| cBrlA-F/R | ATGGACGAGCTGTACAAGATGCAGTTTGAGTCGTCGAGT / CTGCAGGTCG ACGGATCCTCAGTAATCTTCGTGCTTCTCAG | Cloning *brlA* cDNA (1181 bp) for fusion to *GFP* |
| cAbaA-F/R | ATGGACGAGCTGTACAAGATGTCTTCACTATACGCTCCCAGACCCAT / CTG CAGGTCGACGGATCCTCACCAACCGGCCGCAACAGCAACG | Cloning *abaA* cDNA (2706 bp) for fusion to *GFP* |
| qBrlA-F/R | GACCAGTTCAACAGACAAG / CAGTAATCTTCGTGCTTCTC | qPCR assessing *brlA* transcript |
| qAbaA-F/R | GCAAGTCTCCAGCCATAT / CTCCTCTTCGTCATAGTAGTC | qPCR assessing *abaA* transcript |
| q18S-F/R | TGGTTTCTAGGACCGCCGTAA / CCTTGGCAAATGCTTTCGC | qPCR assessing 18S rRNA |
| BrlAup-F/R | TGGGCCCGGCGCGCCGAATTCGGGGGAGAGAGACTATGGAAT / TGGCTGC AGGTCGACGGATCCGGTAAAGGCTGGTGAACAAG | Cloning *brlA* 5′-end (1539 bp) for targeted gene deletion |
| BrlAdn-F/R | GACCCATGGCTCGAGTCTAGACAATGTCAAGCCTGAACCTG / GGTGGTGGT GGCTAGCGTTAACCCTCCTCGTAAAAGATAGCGT | Cloning *brlA* 3′-end (1689 bp) for targeted gene deletion |
| AbaAup-F/R | TGGGCCCGGCGCGCCGAATTCAGGGCAAGTGTTAGTTGAAGGT/ TGGCTGC AGGTCGACGGATCCAATGCGTGTATGTGGTGTATGTG | Cloning *abaA* 5′-end (1519 bp) for targeted gene deletion |
| AbaAdn-F/R | GACCCATGGCTCGAGTCTAGACAGACAGGCACACAAGGATAC / GGTGGTGG TGGCTAGCGTTAACTTGGAGAAGGAGTCAAACAGGT | Cloning *abaA* 3′-end (1740 bp) for targeted gene deletion |
| BrlAfl-F/R | *GGGGACAAGTTTGTACAAAAAAGCAGGCT*CAATTCACCGATGATAAACACC / *GGGGACCACTTTGTACAAGAAAG CTGGGT*CTGCTGCTCACAAAGACACC | Cloning full-length *brlA* (4141 bp) for *brlA* complementation |
| AbaAfl-F/R | *GGGGACAAGTTTGTACAAAAAAGCAGGCT*CTGAGCGTTAGCGACCTGTTG / *G GGGACCACTTTGTACAAGAAAG CTGGGT*AATCAAGGAAAGTTTGCCCATC | Cloning full-length *abaA* (5536 bp) for *abaA* complementation |
| pBrlA-F/R | CTCTTGTTCACCAGCCTTTAC / ATCGCACTTGTCATCCATCT | PCR detecting *brlA* |
| pAbaA-F/R | CTTGTTCTTGCTTCCCGTCAC / TCCTCTTCGTCATAGTAGTCCTCC | PCR detecting *abaA* |
| spBrlA-F/R | GCGATTGATTGCCAAACTCC / CGAGCCTCCTCCTTCTTTCC | Southern probe of *brlA* (295 bp) |
| spAbaA-F/R | ATCGGAGGGTGCCAAAGTC / ACGGGAAGCAAGAACAAGG | Southern probe of *abaA* (353 bp) |

* The underlined regions denote the restriction enzyme sites for the *brlA* or *abaA* cDNA (*Bam*HI) fusion to *GFP* and the deletion of *brlA* or *abaA* (*Eco*RI/*Bam*HI and *Xba*I/*Hpa*I). The underlined and italicized regions are the recognition fragments for the gateway exchange in each complementary plasmid.
